# Valuation of agro-industrial wastes as substrates for heterologous production of α-galactosidase

**DOI:** 10.1186/s12934-018-0988-6

**Published:** 2018-09-03

**Authors:** María-Efigenia Álvarez-Cao, Agustín Rico-Díaz, María-Esperanza Cerdán, Manuel Becerra, María-Isabel González-Siso

**Affiliations:** EXPRELA Group, Centro de Investigacións Científicas Avanzadas (CICA), Facultade de Ciencias, Universidade da Coruña, 15071 A Coruña, Spain

**Keywords:** α-Galactosidase, Cheese whey, Beet molasses, *Saccharomyces cerevisiae*, *Kluyveromyces lactis*

## Abstract

**Background:**

The recycling of agro-industrial wastes is at present limited by the availability of efficient and low-cost enzyme cocktails. The use of these materials as culture media to produce the enzymes can contribute to the profitability of the recycling process and to the circular economy. The aim of this work is the construction of a recombinant yeast strain efficient to grow in mixed whey (residue of cheese making) and beet molasses (residue of sugar manufacture) as culture medium, and to produce heterologous α-galactosidase, an enzyme with varied industrial applications and wide market.

**Results:**

The gene *MEL1*, encoding the α-galactosidase of *Saccharomyces cerevisiae*, was integrated (four copies) in the *LAC4* locus of the *Kluyveromyces lactis* industrial strain GG799. The constructed recombinant strain produces high levels of extracellular α-galactosidase under the control of the *LAC4* promoter, inducible by lactose and galactose, and the native *MEL1* secretion signal peptide. *K. lactis* produces natively beta-galactosidase and invertase thus metabolizing the sugars of whey and molasses. A culture medium based on whey and molasses was statistically optimized, and then the cultures scaled-up at laboratory level, thus obtaining 19 U/mL of heterologous α-galactosidase with a productivity of 0.158 U/L h, which is the highest value reported hitherto from a cheap waste-based medium.

**Conclusions:**

A *K. lactis* recombinant strain was constructed and a sustainable culture medium, based on a mixture of cheese whey and beet molasses, was optimized for high productivity of *S. cerevisiae* α-galactosidase, thus contributing to the circular economy by producing a heterologous enzyme from two agro-industrial wastes.

**Electronic supplementary material:**

The online version of this article (10.1186/s12934-018-0988-6) contains supplementary material, which is available to authorized users.

## Background

Agro-industrial activities generate huge amounts of wastes susceptible to be transformed in culture media for bio-productions with high added value, thus contributing to the circular economy. One outstanding bio-production is the microbial fermentation to ethanol [1, 2] or other biofuels [3] as an alternative to current threat of fossil fuels depletion and growing environmental concerns associated to their massive consumption. The production of heterologous proteins, including enzymes, using microbial cell factories is also remarkable [4].

α-Galactosidases or d-galactoside galactohydrolases (EC. 3.2.1.22) catalyze the hydrolysis of α-(1,6) bonds of galactose residues in galacto-oligosaccharides (melibiose, raffinose and stachyose) and complex galactomannans. Under suitable conditions, α-galactosidases can also catalyze transglycosylation reactions. The α-galactosidase from *Saccharomyces cerevisiae* (ScAGal), encoded by the *MEL1* gene (GeneBank X03102), is a secretory and highly glycosylated protein of 471 amino acids, whose structure has been solved by X-ray crystallography [5]. α-galactosidases show a great variety of uses in the pharmaceutical and food industries such as treatment of Fabry disease [6], conversion between the AB0 blood groups [7], improvement of the separation of sucrose from beet [8], reduction of the content of non-digestible oligosaccharides of legume-derived food products [9] or dietetic supplement [10]. Moreover, the activity of the enzyme α-galactosidase, in synergy with other enzymes, has been reported to facilitate microbial ethanol production from agro-industrial wastes like molasses, bagasse and others [11–14].

The fermentation of agro-industrial waste materials to biofuels is hitherto still a challenge that requires the development of efficient and low-cost enzyme cocktails to be profitable [11]. The use of residues as culture media to produce the required enzymes can help with the economy of the process. Whey is a sub-product of cheese making, rich in the milk sugar lactose, that is produced in increasingly high amounts world-wide [15], while molasses are very abundant sub-products of sugar manufacture, rich in sucrose and raffinose [16, 17]. Large research has been performed aimed to the biotechnological use of these waste materials, thus avoiding their polluting impact, being a main destine their fermentation to bioethanol [1]. An increase in yield has been reported by mixing both sub-products [15, 18–20].

*Kluyveromyces lactis* and *S. cerevisiae* are two food-grade yeasts widely used in biotechnology [21, 22]. Apart from other advantages for heterologous protein production, *K. lactis* is able to use a greater variety of carbon sources than *S. cerevisiae*, being well documented the case of lactose, i.e., the ability to metabolize lactose of the former and the inability of the second species, while both can use sucrose [23, 24]. Due to its special features*, K. lactis* has been used successfully as a cell factory for recombinant protein secretion in large-scale cultures; however, the use of agro-industrial wastes such as whey-molasses mixed media as substrates has not been reported [21]. Mixtures of molasses or bagasse and milk or cheese whey have been used for ethanol production by *Kluyveromyces* sp. [18–20], but not for recombinant protein production.

With the aim to develop a sustainable and cheap system for production of ScAGal, in this paper we describe the work performed to construct recombinant *K. lactis* strains that secrete high levels of ScAGal and to optimize a culture medium based on two agro-industrial waste materials, cheese whey and beet molasses.

## Methods

### Strains and other materials for recombinant DNA techniques

The *K. lactis* strains GG799 {Matα [pGKl1 +]} (New England, Biolabs) and NRRL-Y1140 {Matα} [25] were chosen for the integration and expression of the gene encoding the fusion protein of interest (*MEL1*His). *Escherichia coli* XL1-*Blue* [*recA1 endA1 gyrA96 thi*-*1 hsdR17 supE44 relA1 lac [F’proAB lacIqZDM15 Tn10 (Tetr)]*] (Stratagene Cloning Systems) was used as host for standard recombinant DNA techniques [26]. The plasmid YEp*MEL1*His [amp^r^ ori 2μ *MEL1*His *TRP1*] [27], was used as template to amplify the gene fusion.

Phusion High-Fidelity DNA Polymerase (for gene amplification), DreamTaq polymerase (for colony PCR analysis), and the DNA purification kits GeneJEt Gel Extraction Kit and GeneJET Plasmid Miniprep Kit, were used following the specifications of the supplier (Fisher Scientific).

Low salt LB (1% bactotryptone, 0.5% yeast extract, 0.5% NaCl) and YPD (1% yeast extract, 2% peptone, 2% glucose), were used as culture media for growth and maintenance of bacteria and yeasts, respectively. Low salt LB was supplemented with 100 mg/L ampicillin (Sigma Aldrich) for the propagation of plasmids in bacteria. YCB medium (30 mM Phosphate buffer, pH 7, 1.17% yeast carbon base) supplemented with 5 mM acetamide (Sigma Aldrich) was used for the selection of transformant yeasts. To solid media, 2% agar was added. All the media components were sterilized by autoclave at 121 °C for 20 min, except ampicillin and acetamide that were filtered through 0.22 μm microfiltration membrane (Sartorius AG).

### Construction of recombinant *K. lactis* strains

**Fig. 1:**
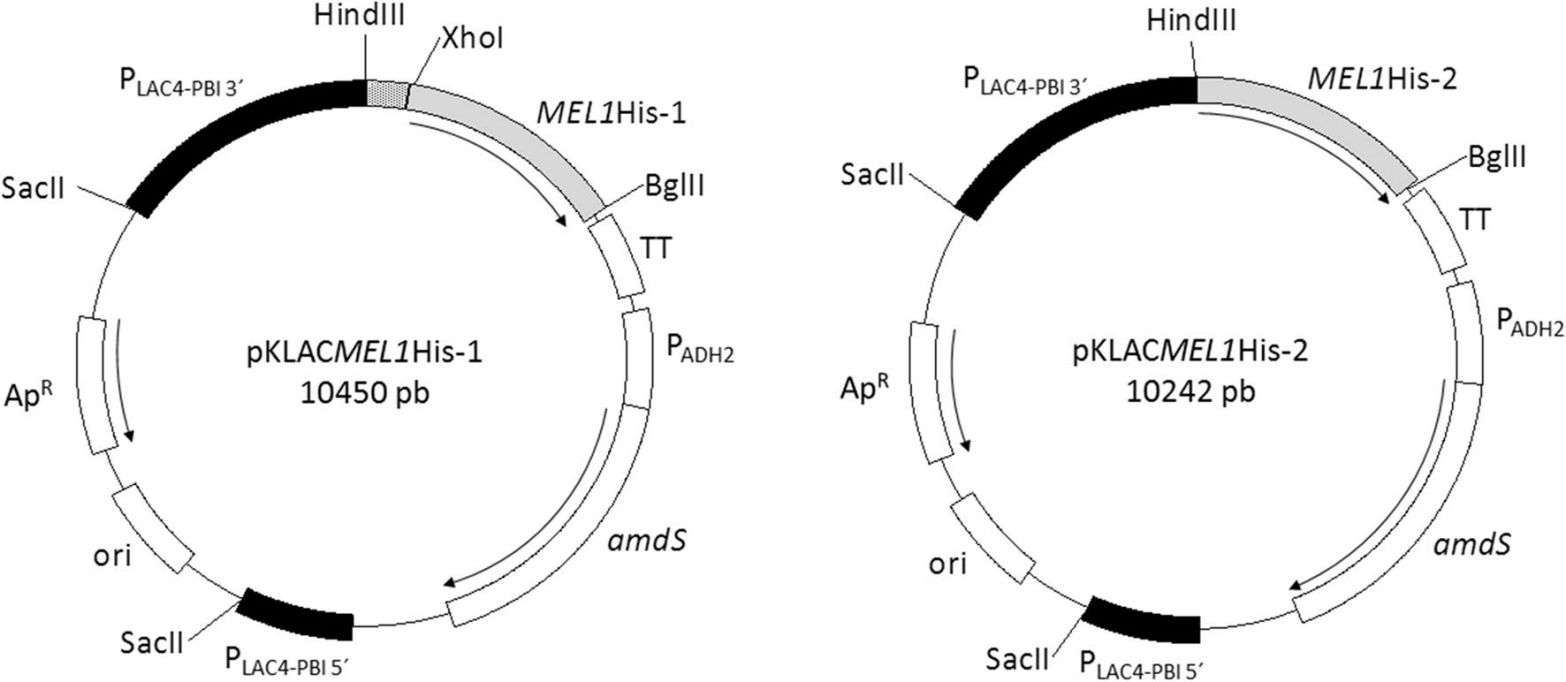
Physical maps of the plasmids constructed to generate the recombinant *K. lactis* strains. Dotted box = *K. lactis* α-MF sequence

**Fig. 2:**
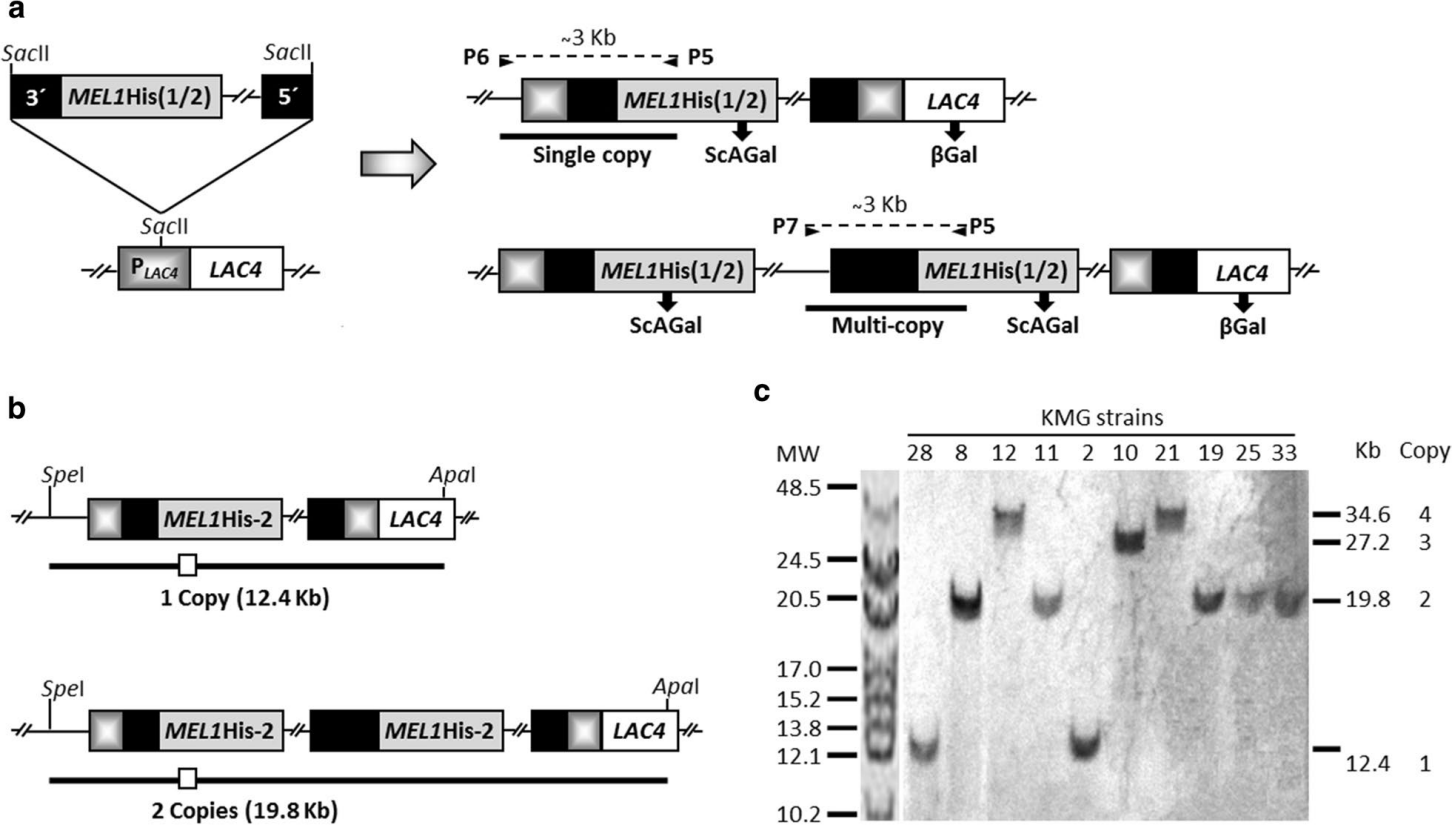
Directed integration of *MEL1*His gene in the *K. lactis LAC4* chromosomal locus. **a** The linearized expression cassette (*MEL1*His-1 or *MEL1*His-2) is guided by the 3´and 5´ends of P_*LAC4*-*PBI*_ (black boxes) to the *LAC4* promoter region that is reconstructed after the integration. P6 primer anneals upstream P_*LAC4*_ of the original strain and P7 primer anneals upstream P_*LAC4*-*PBI*_ inside the expression cassette. Primer pairs P6-P5 and P7-P5 were used to analyze if the integrations were single or multiple copy, respectively. **b** During Southern blot analyses, the genomic DNA was digested with *Spe*I and *Apa*I, and bands separated by electrophoresis before Southern transference. The probe (white box) when hybridizes with single copy bands reveals a fragment of 12.4 Kb that increases in 7.4 Kb per additional cassette integrated in multicopy strains. **c** Immunological detection and colour reaction allowed to determine the number of copies of the integrated cassette in KGM strains. MW, molecular weight marker (Kb)

Two different constructions with the gene *MEL1*His, i.e. *MEL1* that encodes ScAGal (Uniprot P04824) fused to a Poli-His sequence by the C-terminal, were cloned into the integrative *K. lactis* expression vector pKLAC-1 (New England Biolabs). The complete *MEL1*His ORF with and without the N-terminal *K. lactis* α-MF domain sequence (encoding 18 amino acids) were amplified with the primer pairs P1/P3 and P2/P3, respectively (Table 1). P1 was designed to encode the excision site of the endoprotease Kex, and thus assure the correct processing of the fusion protein with the signal peptide of the *K. lactis* α-MF. P2 contains the *Hin*dIII site directly in frame with the ORF *MEL1*His, excluding the α-MF domain but with the native signal sequence of *MEL1*. The PCR products were purified and cloned between the *Xho*I-*Bgl*II and *Hin*dIII-*Bgl*II sites of pKLAC-1, giving rise to pKLAC*MEL1*His-1 and pKLAC*MEL1*His-2, respectively (Fig. 1). The recombinant plasmids were verified by sequencing (Servizos de Apoio á Investigación, Universidade da Coruña) with primers P4 and P5 (Table 1). Both plasmids were linearized with *SacII*, to obtain the expression cassettes that, after being purified, were integrated by homologous recombination in competent cells of *K. lactis* GG799 and Y1140 that were transformed by the lithium acetate procedure [28]. The transformants were selected in YCB-acetamide plates after incubation at 30 °C for 4 days. Recombinant colonies were checked by PCR [29] with the primers P5, P6 and P7 (Table 1) that anneal inside and outside the expression cassette, to confirm that the integration in single and multiple copy was correct (Fig. 2a).

**Table 1:** Oligonucleotides used in this study

Primer	Sequence (5′ to 3′)^a^	Strand	Applied strategy
P1	CCGCTCGAG*AAAAGA*GTGTCTCCGGTTACAATGGCCTTGG	F	*Xho*I cleavage site
P2	CCCAAGCTTATGTTTGCTTTCTACTTTCTCAC	F	*Hin*dIII cleavage site
P3	GGAAGATCTTCA**GTGGTGGTGGTGGTGGTG**AGAAGAGG	R	*Bgl*II cleavage site
P4	GTGGTTGCTTTATTGAATGGAG	F	Sequencing
P5	TTCAAGTAGTCAACGCGGTTA	R	Sequencing, integration
P6	ACACACGTAAACGCGCTCGGT	F	Single copy integration
P7	CAGTGATTACATGCATATTGT	F	Multi-copy integration
P8	TCAAGCCTCTGTCATCGCAAT	F	*MEL1* probe (qPCR)
P9	TCTCTTCCAAGGTCGTGTTCATT	R	*MEL1* probe (qPCR)
P10	AAATGGAAGACAACCCGCC	F	*TAF10* probe (qPCR)
P11	TTACGAATTTCTGTGTAGCAAGAGC	R	*TAF10* probe (qPCR)
P12	ACACCTGTGCTGGATATCCTG	F	*MEL1* probe (*Southern*)
P13	GATCATTCCAACCACCAACAC	R	*MEL1* probe (*Southern*)

*F*, forward strand; *R*, reverse strand

^*a*^Engineered restriction sites are underlined, Poly-His tag is indicated in blond and Kex protease cleavage site in italics

### Determination of the number of copies of the integrated cassette

qPCR and *Southern blot* analysis were carried out to determine the copy number of the integrated exogenous DNA. Genomic DNA isolation from yeasts was performed by the method described in [30]. Amount and quality of DNA were estimated by 0.7% agarose gel electrophoresis and spectrophotometry (Biospectrometer Kinetic Eppendorf). Samples with a ratio 260 nm/280 nm equal or higher than 1.8 were selected. qPCR was performed using an Eco Real-Time PCR System with software version 4.0.7.0 (Illumina, Inc., San Diego, California, USA). The threshold cycle (Ct) was determined by the ‘Fit Points Method’ in the software. The primers were designed with Tm 60.59–60.49 °C to synthesize amplicons of 203 bp. Reaction mixture was prepared with KAPA SYBR FAST Kit (Kapa Biosystems) that uses SYBR Green as fluorophore. Serial dilutions of 10 ng/μL template DNA (three independent extractions) were amplified with specific primers for each gene (Table 1). The reaction was developed in 48-well optic plates Ecoplate (Bibby Scientific LTd) sealed with Box Seals Adhesive (Illumina). Thermal profile consisted of one step of 5 min at 95 °C followed by 40 cycles of 3 s at 95 °C and 3 s at 60 °C, fluorescence was recorded at the end of the elongation step. The amplification period was followed by analysis of the melting curve with temperature gradient 0.1 °C/s from 55 °C to 95 °C to avoid amplification of unspecific products. Relative quantification of copy number was according the 2^−ΔΔCt^ method with the equation [31]: relative amount of target gene = (1 + E)^–ΔΔCt^ where, ΔΔCt = ΔCt target gene − ΔCt calibrator gene; ΔCt = Ct target or calibrator gene − Ct reference gene; E = PCR efficiency. Results are presented as the ratio between *MEL1*His amount, using as calibrator a single copy strain identified by colony PCR analysis, and the amount of endogenous reference gene *TAF10* that is constitutive, not affected by *MEL1*His. PCR efficiency was calculated from the slopes of standard curves generated by lineal regression of average values of Ct versus log_10_ of template DNA concentration from the calibrator strain [32].

The kit DIG DNA Labelling and detection Kit (Roche) was used for Southern blot assays. The probe was prepared using the primers P12/13 (Table 1) to obtain an amplicon of 423 bp that was purified and labelled with digoxigenin. Genomic DNA (3 ng/μL) was incubated with *Spe*I and *Apa*I at 37 °C overnight. Digested samples and GeneRuler High Range DNA LAdder (Thermo Scientific) were loaded, with buffer containing GelGreen Nucleic Acid Gel Stain (Biotium), in a 0.4% agarose gel and electrophoresed. Separated fragments were transferred to a positively charged nylon membrane Hybond-N+ (GE Healthcare) using the protocol as per [26], and hybridization was performed at 52 °C for 12 h with the labelled probe denatured at 80 °C for 3 min. Then, the membrane was washed 10 min at 52 with 2X SSC (0.6 M NaCl, 60 mM sodium citrate, pH 7) containing 0.1% SDS, and a second wash of 10 min at room temperature. After immunological detection and colorimetric reaction, performed as described in the kit protocol, the membrane was photographed, and the visualized fragments analyzed with the program Molecular Imager Gel Doc XR+ (BioRad).

### Culture media and conditions for ScAGal expression in recombinant *K. lactis* strains

Culture media used to induce the expression of the heterologous protein ScAGal in the *K. lactis* recombinant strains constructed in this work were: YPG (1% yeast extract, 2% peptone, 2% galactose), YPL (1% yeast extract, 2% peptone, 2% lactose) and YPW (1% yeast extract, 2% peptone, 2% cheese whey). Also, a culture medium based in the mixture of the food industry subproducts cheese whey and beet molasses was used to optimize the production of the heterologous protein ScAGal, as described below. Cheese whey (concentrated ultrafiltration permeate) and beet molasses were provided by QUEIZUAR, SL (Bama, A Coruña, Spain) and AB Azucarera Iberia, formerly Azucarera Ebro (Spain), respectively. Molasses were diluted in distilled water ratio 1:1 (v/v) to facilitate handling and, before autoclave, both molasses and cheese whey were centrifuged at 10,000 rpm for 15 min to remove solid impurities. Sugars present in molasses and cheese whey were identified and quantified by HPLC, using as standards mixtures of raffinose, sucrose, glucose, galactose and fructose in the case of molasses; and mixtures of lactose, glucose and galactose in the case of cheese whey. Both standards were prepared with five points from 4 to 0.06 mg/mL of each analyte.

To prepare the inoculum of the cultures, one isolated *K. lactis* colony was grown in YPD at 30 °C and 250 rpm until reaching an OD at 600 nm of 10. This pre-culture was used to inoculate 100 mL Erlenmeyer flasks containing 20 mL of YPG, YPL or YPW up to an initial optical density at 600 nm (OD_600_) of 0.5 (30 °C, 250 rpm). For the cultures with the optimized medium, an initial inoculum of 10 OD_600_ was used, keeping the rest of the here described conditions.

### Heterologous expression of ScAGal in recombinant *K. lactis* strains

Cultures of selected transformants isolated in YCB-acetamide were initially performed in 96-well microtiter plates (TC Plate 96 Well, Sarstedt) with 200 μL YPD per well and incubated at 30 °C and 300 rpm. After 24 h of incubation, 20 μL of each well were used to inoculate a second plate with 200 μL YPG per well, to induce the expression under the same incubation conditions. After 72 h of incubation, the plates were kept at 4 °C to favor cell decanting and then analyze extracellular α-galactosidase activity with the cell-free supernatant. In each case, the plates contained three cultures of a recombinant strain with single copy integration. Then, the candidate with single copy integration and those with multiple copy integration that showed the highest activity were chosen for the cultures in shaken flasks with YPG as described above. Samples were taken at predetermined time intervals to measure growth (biomass) and enzymatic activity.

Biomass was measured as OD_600_ with a UV–Visible spectrophotometer (Biospectrometer Kinetic Eppendorf). Culture samples were centrifuged (5000 rpm, 5 min) and supernatants were used to measure extracellular activities of heterologous α-galactosidase and native invertase, whereas the cell pellet was permeabilized with 15% chloroform and 0.01% SDS to measure native β-galactosidase.

### Optimization of ScAGal production by recombinant *K. lactis* strains

The chosen *K. lactis* recombinant strain was first cultured in YPL and YPW. Then, the surface response methodology (SRM) was applied to maximize ScAGal production in a medium composed by cheese whey (concentrated ultrafiltration permeate) and beet molasses. A factorial experimental design 3^2^ allowed to study the influence, individual and combined, of the factors (independent variables) cheese whey and beet molasses concentration, on the response (dependent variable) that is quantified as the extracellular α-galactosidase activity of the performed cultures under the assayed conditions. Each factor was added at the concentration determined by the experimental design as percentage of total sugars, and all the cultures were supplemented with 1% yeast extract to provide nitrogen source. Real and coded values for the factors at three different levels, − 1, 0, + 1, being 0 the central point of the experimental domain, are shown in Table 2. A second order quadratic model was used to correlate the response or dependent variable with the independent variables or factors, and the optimum point of the experimental domain was obtained solving the regression equation and analyzing the contour response surface graphic. An ANOVA was applied, with confidence intervals of 95%, to determine the statistical significance of the effects of the factors.

**Table 2:** Experimental domain and codification of the independent variables in the factorial design 3^2^

Real values	Coded values^a^		
	− 1	0	1
Beet molasses (%), *X*_1_	6	7.5	9
Cheese whey (%), *X*_2_	2	3	4

^a^*x*_*i*_= (*X*_*i*_ *− X*_0_)/∆*X*_*i*_, *i* = 1, 2; where *x*_*i*_ and *X*_*i*_ are the coded and real values of the independent variable *i*, X_0_ is the real value of the independent variable *i* at the central point, and ∆*X*_*i*_ is the step change value

### Laboratory scale bioreactor

The selected recombinant *K. lactis* strain was cultured in a Biostat-MD 2 L (Braun-Biotech) bioreactor with a 1 L working volume of the optimized production medium. The bioreactor was sterilized by autoclave at 121 °C for 30 min, inoculated at an initial OD_600_ of 10, and aseptically supplemented with 300 mg/L of ampicillin. Culture temperature was 30 °C, air flow rate 2 L/min and stirring 300 rpm, conditions maintained during 120 h. Cell biomass was measured as dry weight from 5 mL culture samples, centrifuged at 5000 rpm for 5 min, washed with distilled water, centrifuged again and dried in oven at 105 °C until constant weight. The supernatant was used to measure extracellular α-galactosidase activity and the conversion of substrates into secondary metabolites. Sugar consumption and metabolites production was analyzed by HPLC using as external standard raffinose, sucrose, glucose, galactose, fructose, glycerol and ethanol at five concentrations ranging from 4 to 0.06 mg/mL.

### Enzymatic activity assays

α-galactosidase and β-galactosidase activities were measured at 40 °C using as substrates 10 mM PNPG in McIlvaine buffer pH 4 [33] and 13 mM ONPG in Z buffer [34], respectively. Both substrates were supplied by Sigma Aldrich. At two consecutive time intervals, the reaction was stopped with 0.5 M Na_2_CO_3_ and released *p*-nitrophenol and *o*-nitrophenol were quantified at 400 nm (ε = 18.2 L/mmol/cm) or 420 nm (ε = 4.5 L/mmol/cm), respectively. Invertase activity was measured with 100 mM sucrose as substrate in 50 mM acetate buffer pH 5 for 10 min at 40 °C, the reaction was stopped by boiling (95 °C, 5 min) and the increment in reducing sugars was quantified by the DNS method [35]. Assays were performed in triplicate and spectrophotometric readings were performed in flat bottom microtiter plates with Synergy H1 Hybrid Multi-Mode Reader (BioTEk). One enzymatic unit (U) is defined as the amount of enzyme that releases one μmol of product per min under the assay conditions.

### HPLC analysis

A Sugar Pack Waters (6.5 mm × 300 mm) column with Refractive Index Detector (Cienytech) was used. The samples were clarified with the cartridges HyperSep Silica SPE Column (Fisher Scientific). Running conditions were, column temperature 80 °C, detector temperature 37 °C, sensitivity 32 and using 100 μM EDTA-Calcium (Sigma Aldrich) at a flow rate of 0.5 mL/min as mobile phase. Eluted compounds were identified and quantified using the corresponding external standards. To each sample and external standard 1 mg/mL sorbitol was added as internal standard [36].

### Statistical analysis

Statistical data treatment and graphics drawing was performed with the program StatGraphics Plus version 5.1. *p*-values were calculated with Student’s *t*-tests and results were considered significant for *p*-values less than or equal to 0.05.

## Results and discussion

### Construction of recombinant *K. lactis* strains

The expression cassettes, linearized by *Sac*II digestion from the plasmids pKLAC*MEL1*His-1 and pKLAC*MEL1*His-2 (Fig. 1), were successfully integrated in the genome of the *K. lactis* strains GG799 and Y1140. The integration occurred, by homologous recombination, in the promoter region of the *LAC4* locus [37]. The *amdS* gene was used as selection marker, this gene encodes the enzyme acetamidase that allows the growth of the transformants in a minimal medium with acetamide as single nitrogen source. The transformants contained either one or several tandem copies of the *MEL1* gene that were expressed under the *LAC4* promoter, while the native *LAC4* locus, that drives the expression of the enzyme β-galactosidase, was reconstructed (Fig. 2a). Single copy and multiple copy integrations were identified by PCR with the primers pairs P6/P5 and P7/P5 (Table 1), respectively. The frequency of multiple copy integration (50-60% of total transformed clones) was similar for the *K. lactis* strains GG799 and Y1140 (Table 3).

**Table 3:** Integration frequency and extracellular ScAGal activity of multiple copy *K. lactis* transformants

Recombinant strain	Integration			ScAGal activity^a^	
	Type	Clones	%	U/mL	Clones
GG799*MEL1*His-1	Single copy	24	51	0.039 ± 0.014	3
	Multi-copy	23	49	0.108 ± 0.001	18
				0.193 ± 0.078	5
GG799*MEL1*His-2	Single copy	14	42	0.167 ± 0.066	3
	Multi-copy	19	58	0.362 ± 0.104	11
				0.741 ± 0.200	8
Y1140*MEL1*His-1	Single copy	15	42	0.005 ± 0.001	3
	Multi-copy	21	58	0.010 ± 0.001	17
				0.020 ± 0.001	4
Y1140*MEL1*His-2	Single copy	13	43	0.112 ± 0.030	3
	Multi-copy	17	57	0.210 ± 0.029	13
				0.372 ± 0.042	4

Each 96-well dishes cultures contained three single copy transformants identified as described in Methods section, which express only one copy of ScAGal. A unit (U) is defined as μmol.min to pH 4 and 40 °C

### Heterologous expression of ScAGal

Table 3 shows the values of extracellular ScAGal activity measured from 96-well microtiter cultures in YPG medium, which allowed to classify the multiple copy transformants in two groups, probably with two or with more than two integrated copies of the *MEL1* gene.

The extracellular ScAGal activity of the strain GG799*MEL1*His-2 was fourfold (4 ± 0.465) higher than that of GG799*MEL1*His-1, whereas the extracellular ScAGal activity of the strain Y1140*MEL1*His-2 was 20-fold (20 ± 2.178) higher than that of Y1140*MEL1*His-1 (Table 3). These results show that the extracellular ScAGal activity was notably higher when the endogenous signal sequence of *S. cerevisae MEL1* was used (*MEL1*His-2 cassette) in comparison with the *K. lactis* α-MF domain sequence (*MEL1*His-1 cassette).

However, in all cases, GG799 was the strain that secreted higher levels of extracellular ScAGal activity regardless of the number of copies of *MEL1* integrated into its genome. Although these results confirm the advantage of the pKLAC-1/*Klactis* GG799 expression system recommended by the manufacturer (*K. lactis* protein expression kit, New England Biolabs) *vs* another wild type strain, such as Y1140, we also demonstrated the interest of carrying out a study of the native signal peptide of the heterologous protein before being replaced by an exogenous signal peptide (*K. lactis* α-MF) to drive its secretion to the culture medium.

**Fig. 3:**
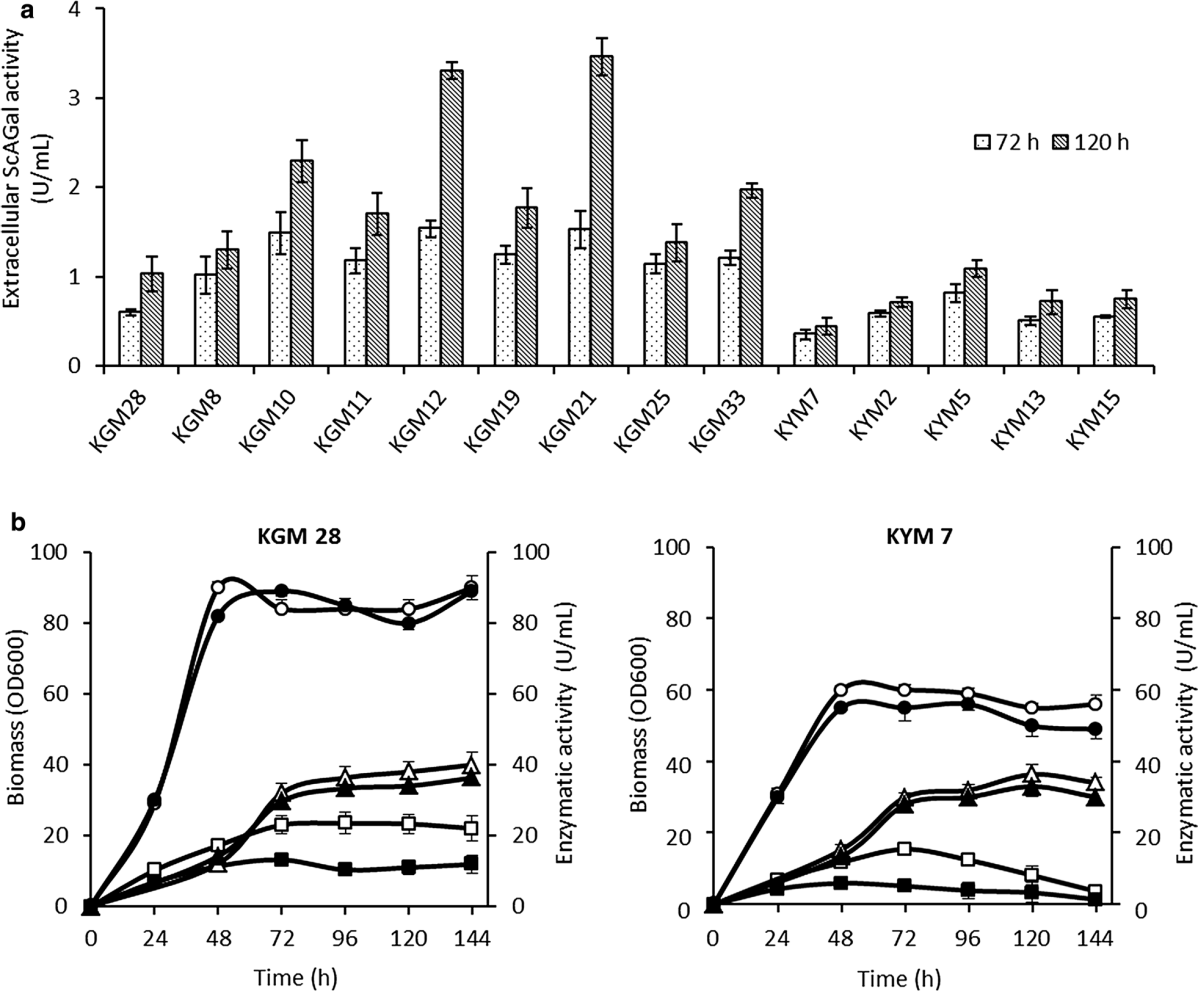
Time course of cultures from the strains KGM and KYM in shaken flasks using YPG medium. **a** Extracellular α-galactosidase activity of selected multicopy transformants. **b** Typical profile of growth (circles), native β-galactosidase (squares) and invertase (triangles) of wild type (empty) *versus* single copy strains (full). KGM28 and KYM7 are single copy transformants with *MEL1His*-2. Data shown are average ± SD, N = 3

The multiple copy transformants of both strains with *MEL1*His-2 showing the highest extracellular activity were selected to analyze the expression of ScAGal at a higher scale in shake flask cultures (Fig. 3a). Higher values of extracellular ScAGal activity were obtained with GG799*MEL1*His-2 transformants (abbreviated KGM from now on) than with Y1140*MEL1*His-2 transformants (abbreviated KYM from now on), which is corroborated by the preliminary results shown above in the same medium. On the other hand, the activity of the native enzymes β-galactosidase and invertase was also measured from single copy transformants of KGM and KYM in comparison with the corresponding wild type strains (Fig. 3b). KGM expressed an average of β-galactosidase activity of 23 U/mL that was maintained until 144 h, while KYM obtained 15 U/mL decreasing abruptly in the same time range. Further, KGM reached the stationary phase at 48 h with a higher cellular biomass and both strains secreted around 30–35 U/mL of invertase. This result also shows the highest production of β-galactosidase by both wild strains, and suggests that integration of the heterologous gene in the *LAC4* promoter negatively affects the expression of the *LAC4* gene, as was confirmed in a chymosin producing strain vs wild strain using proteomic analysis [24]. For all these reasons, KGM was selected as the best *K. lactis* expression system of ScAGal for further research.

### Copy number distribution of *MEL1* in KGM multiple-copy strains

A total of 10 KGM strains were selected for qPCR analysis, eight bearing multi-copy integration and 2 bearing single-copy integration. The single copy strains KGM28 and KGM2, were used as calibrator to generate the standard curves and control of the calibrator, respectively. The vast majority of qPCR data in the literature is derived from the use of relative quantification by employing reference genes, which must be at a similar *concentration* to the target In order to get valid results [32, 38]. The house-keeping gene *TAF10*, TATA Binding Protein (TBP) Associated Factor 10, was shown to be a reliable endogenous control. *TAF10* is a single copy gene similar to *S. cerevisiae YDR167W TAF10* subunit (145 kDa) of *TFIID* and *SAGA* complexes involved in RNA polymerase II transcription initiation and in chromatin modification [39]. Both for the gene *MEL1* and the reference gene *TAF10* they were not curve but straight lines (R^2^ > 0.994) and showed a high amplification efficiency (Additional file 1: Figure S1A). The lineal regression analysis of the ΔCt variation between both genes versus the template DNA concentration gave a slope close to zero (0.0755), which confirms that amplification efficiencies are similar enough to validate the use of the 2^−ΔΔCt^ method [31] (Additional file 1: Figure S1B). Since KGM28 contains one copy of the specific sequence of each gene, the copy number ratio *MEL1/TAF10* for the calibrator is 1 and, therefore, the ΔCt of the calibrator (1.98 ± 0.38) was employed to normalize the copy-number ratio *MEL1/TAF10* for the analyzed strains. Table 4 shows the results of the relative quantification and the copy number estimation by the 2^−ΔΔCt^ method.

**Table 4:** Estimated copy number of *MEL1*His-2 integrative cassette by relative quantification

Strain	Ct_*MEL1*; n_	Ct_*TAF10*; n_	∆Ct_n_^a^ (Ct_*MEL1*; n_ − Ct_*TAF10*; n_)	∆∆Ct (∆Ct_n_ − ∆Ct_c_)	Copy number^b^	
					2^−∆∆Ct^	(1 − E)^−∆∆Ct^
KGM2^c^	22.24 ± 0.43	24.24 ± 0.52	− 1.99 ± 0.41	− 0.01	1.01	1.01
KGM8	16.56 ± 0.29	19.51 ± 0.31	− 3.19 ± 0.24	− 1.20	2.31	2.45
KGM10	19.37 ± 0.32	22.91 ± 0.22	− 3.53 ± 0.34	− 1.55	2.94	3.17
KGM11	16.58 ± 0.45	19.27 ± 0.39	− 2.68 ± 0.43	− 0.70	1.62	1.68
KGM12	17.26 ± 0.12	21.14 ± 0.23	− 3.88 ± 0.23	− 1.89	3.71	3.97
KGM19	19.61 ± 0.20	22.52 ± 0.54	− 2.91 ± 0.21	− 0.92	1.90	1.99
KGM21	17.55 ± 0.62	21.42 ± 0.22	− 3.86 ± 0.47	− 1.88	3.68	4.08
KGM25	19.01 ± 0.16	22.05 ± 0.41	− 3.03 ± 0.44	− 1.05	2.07	2.18
KGM33	18.33 ± 0.32	21.65 ± 0.66	− 3.31 ± 0.12	− 1.28	2.43	2.59

^a^Calculated from three extractions of genomic DNA samples, n = 3 ± SD

^b^∆Ct_c_ (calibrator gene ∆Ct) and E (PCR efficiency) were obtained experimentally of the standard curves calculated from the serial tenfold dilutions of the genomic DNA (10 ng/µL) of the calibrator strain (∆Ct_c_ = 1.98 ± 0.38; E = 1.12; n = 4 ± SD; average values were evaluated by *t*-test, *p*-value < 0.05)

^c^KGM2 was the single copy strain used as control of the calibrator to validate that it is not affected by experimental treatment

The number of copies of the *MEL1*His-2 gene integrated in the KGM strains was also analyzed by *Southern blot* using a specific *MEL1* probe labelled with digoxigenin (Fig. 2b). In this analysis, the number of copies was calculated from the length of the hybridized DNA fragments (Fig. 2c). In addition, the southern blot analysis allowed to exclude the integration of further expression cassettes in other orientations or in other loci. Up to 4 copies of the *MEL1*His-2 gene were integrated in the KGM strains, as determined by qPCR (Table 4) and corroborated by *Southern blot* (Fig. 2c). KGM21 was the strain selected for further research, due to its highest number of integrated *MEL1*His-2 copies (Table 4) and levels of ScAGal extracellular activity (Fig. 3a).

### Optimization of a medium based on cheese whey and beet molasses for extracellular ScAGal production by the strain KGM21

With the aim to develop a sustainable and cheap culture medium for heterologous ScAGal extracellular production, while valuating both wastes of the food industry, mixtures of cheese whey and beet molasses were assayed. As shown above, the strain KGM21 expresses four copies of the gene encoding ScAGal under the *LAC4* promoter that is inducible by lactose. Recombinant ScAGal allows the hydrolysis of raffinose and its use as carbon source for growth. This strain also produces native β-galactosidase and thus is able to use lactose as carbon source for growth. Moreover, KGM21 produces the native enzyme invertase, allowing the use of sucrose as carbon source for growth. HPLC analyses confirmed that lactose (99% w/v) is the major component of cheese whey, while sucrose (60% w/v) followed by raffinose (3% w/v) are the main components of beet molasses, in the lots used in this work.

The extracellular production of ScAGal by KGM21 in media with pure lactose and whey, YPL and YPW respectively, both with a 2% final lactose concentration, was compared. The levels of ScAGal were more than double in the whey medium (3.7 ± 0.15 U/mL) than in the lactose medium (1.4 ± 0.25 U/mL), during 144 h of culture (Additional file 2: Figure S2). In addition, YPW with a 4% final lactose concentration allowed a considerable increase of ScAGal in the same time assayed (7.5 U/mL). Therefore, cheese whey proved to be a good substrate for ScAGal production by KGM21.

Then, beet molasses were assayed as an additional carbon source for ScAGal production by KGM21, mixed with cheese whey, by means of an experimental factorial design 3^2^ that was composed of 12 experiments with three replicates of the central point (Table 5). We selected, as values of the two variables at the central point of the design, a 3% of whey based on the data observed above and a 7.5% of molasses because *Kluyveromyces* sp. at higher concentrations may exhibit catabolite repression in these mixtures and not be able to consume lactose [20]. Since nitrogen fraction of whey and molasses is low [15], the culture media were supplemented with yeast extract. Commercial yeast extract can be replaced by an autolysate of the yeast biomass grown in the cultures [40], and thus this supplement does not represent an economic load. Cultures were inoculated at a DO_600_ = 10 (1 mg dry matter/mL) to favor the consumption of all available sugars.

**Table 5:** Experimental matrix of the 3^2^ factorial design and results obtained of each experiment and predicted from RSM

Exp no.	Coded values		Real values^a^		(U/mL)^b^	
	*x*_1_	*x*_2_	*X*_1_	*X*_2_	Observed	Estimated (*Y*)
1	− 1	− 1	6	2	3.813	3.84963
2	0	− 1	7.5	2	5.286	5.20958
3	1	− 1	9	2	1.945	1.98479
4	− 1	0	6	3	4.571	4.88225
5	0	0	7.5	3	5.945	5.70596
6	1	0	9	3	1.64	1.94492
7	− 1	1	6	4	5.165	4.81712
8	0	1	7.5	4	4.412	5.10458
9	1	1	9	4	1.152	0.807292
10	0	0	7.5	3	6.363	5.70596
11	0	0	7.5	3	5.324	5.70596
12	0	0	7.5	3	5.808	5.70596

^a^X_1_, beet molasses (%); X_2_, milk whey (%)

^b^Extracellular ScAGal activity (μmol min/mL) to pH 4 and 40 °C

**Fig. 4:**
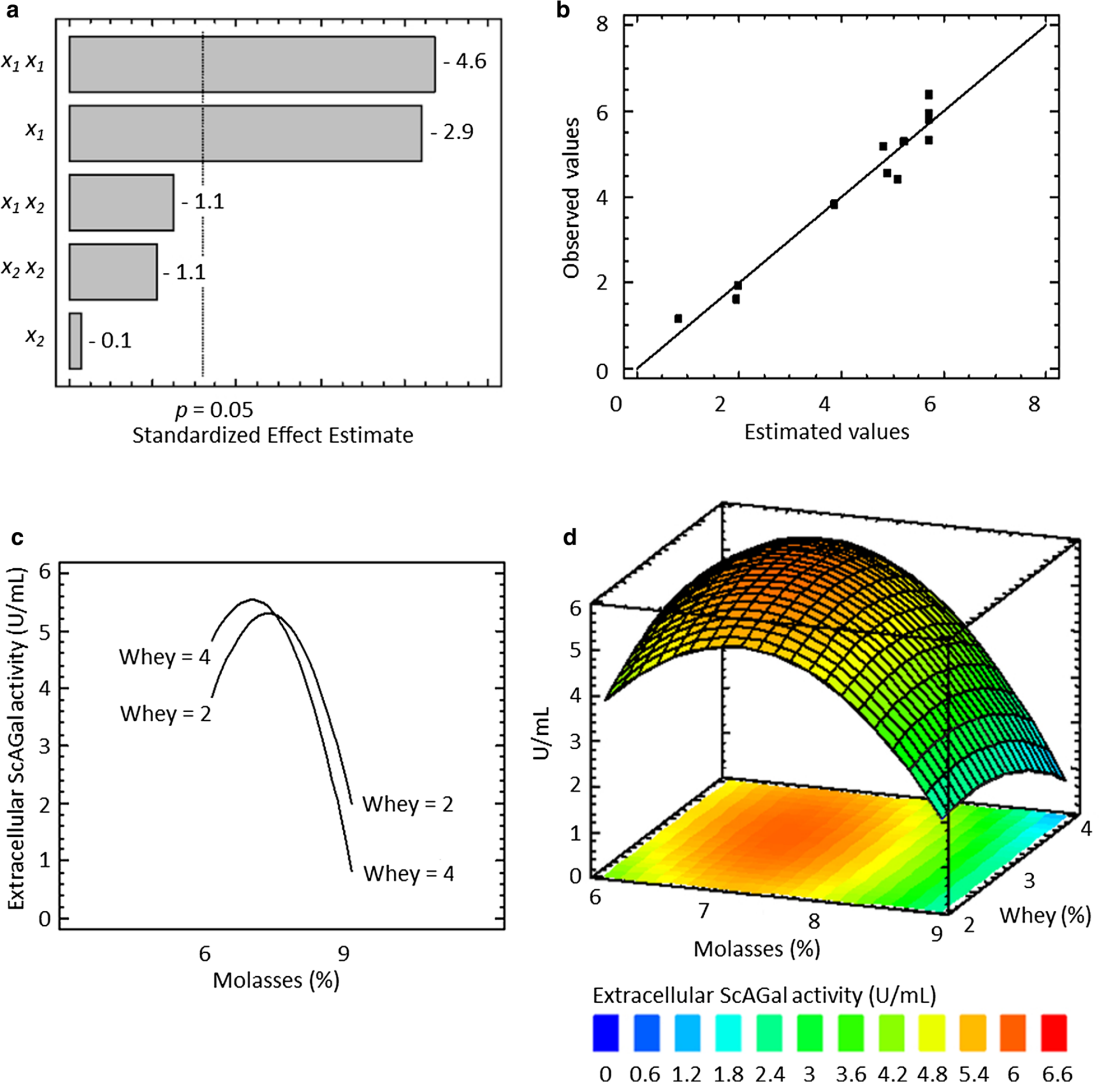
Optimization of a medium based on cheese whey and beet molasses for extracellular ScAGal production by the strain KGM21 using RSM. **a** Pareto chart of the effects of the independent variables on the response at the 95% significance level (vertical line). **b** Observed versus adjusted values by the regression model. **c** Interaction molasses-whey effect, where the factor molasses ranges from − 1 to + 1 and the factor whey is maintained constant at the value + 1 (up line) and − 1 (down line). **d** Response surface-contour plot of the combined effect of molasses and whey indicating the estimated maximum value

The results of extracellular ScAGal activity obtained of each experiment and predicted from RSM are shown in Table 5. Multiple regression analysis was performed to fit the response function to the experimental data, which resulted in the following quadratic polynomial equation: *Y* = − 57.09 + 15.37*x*_1_ + 5.92*x*_2 _− 1.019*x*_1_*x*_1 _− 0.357*x*_1_*x*_2 _*− *0.549*x*_2_*x*_2_, where *Y* is the extracellular ScAGal activity (U/mL) and *x*_1_ and *x*_2_ are the coded values for the concentrations of beet molasses and cheese whey, respectively. The *R*^2^ statistic indicated that the model explains the 95.51% of the variability of the response (*Y*) and the rest of the total variance is attributed to deviations different from the experimental factors. The “lack of fit” was not statistically significant (*p* value = 0.338), which confirms that the model is suitable to predict the extracellular production of ScAGal into the experimental domain used (Additional file 3: Table S1). The Pareto chart of effects of each independent variable on the response-extracellular ScAGal activity shows that the concentration of beet molasses was the highest negative effect on the response (Fig. 4a). A good correlation between the experimental results and the values predicted from the model was confirmed (Fig. 4b), so that the statistically not significant effects were not excluded. Also, it is observed an inflection point of extracellular ScAGal activity, from which and to both sides, it decreases when molasses concentration varies up or down, with independence of whey concentration (Fig. 4c). Finally, the response surface graphical representation and its corresponding contour plot allowed to determine the optimal point, the conditions that yield the highest value of the response (Fig. 4d). In this case, the theoretical optimal conditions were found close to the central point of the experimental domain: 7% beet molasses and 3% cheese whey, yielding a value predicted from the model of 5.95 U/mL of extracellular ScAGal activity.

### Scale-up of extracellular ScAGal production by the strain KGM21

**Fig. 5:**
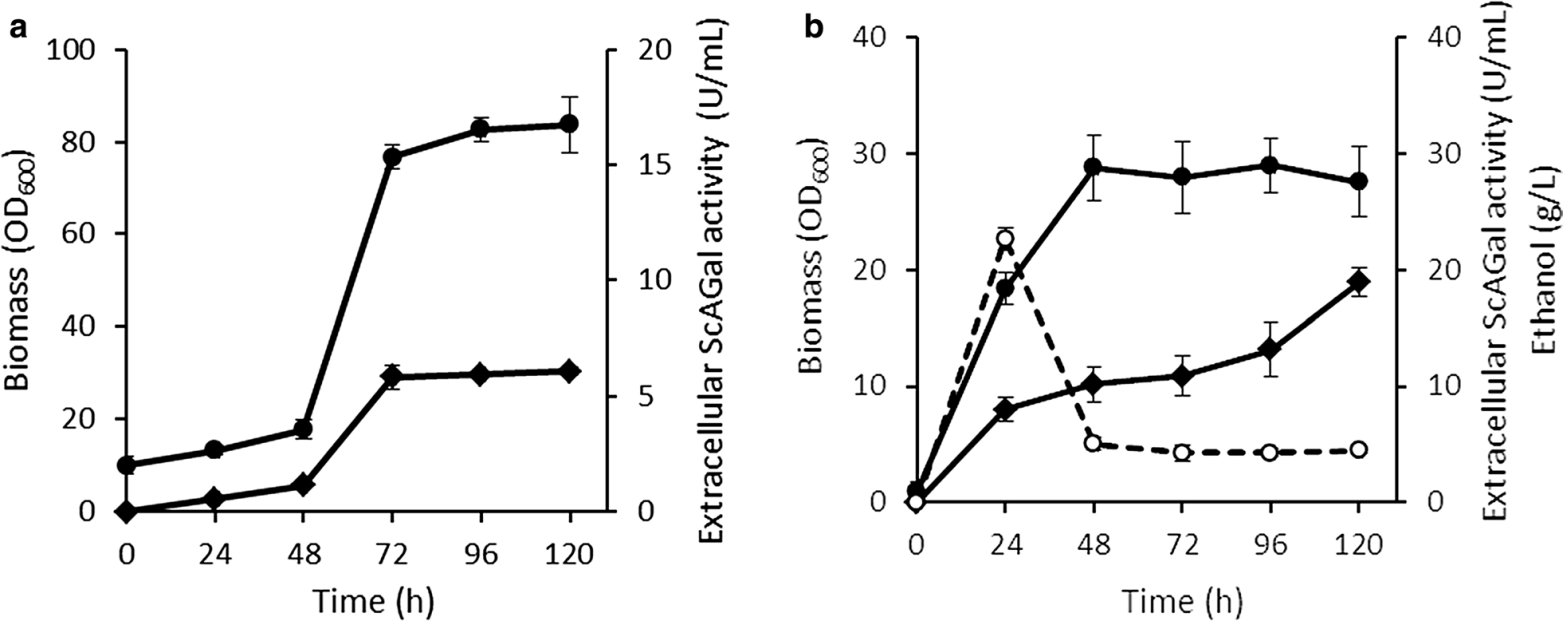
KGM21 culture in shaken flasks (**a**) and bioreactor (**b**) with the optimized culture medium of molasses and whey. Biomass (full circles), extracellular α-galactosidase activity (full diamonds), ethanol (empty circles). Data shown are average ± SD, N = 3

Using the culture medium optimized as described above, the production of extracellular ScAGal by KGM21 was scale-up to shake flask and bioreactor levels. The substrates present in the optimized medium were 74 g/L sucrose, 13 g/L fructose, 12 g/L glucose, 10 g/L lactose, 6 g/L raffinose and 1 g/L galactose, which were identified and quantified by HPLC. Figure 5a shows that in shake flask cultures, extracellular ScAGal production increased in parallel to cellular growth up to reaching the stationary phase at 72 h, the average enzyme activity value of 5.83 U/mL was maintained during the stationary phase and is concordant with the model prediction. Figure 5b shows that in bioreactor cultures KGM21 arrived at the stationary phase in 48 h showing an extracellular ScAGal activity value of 10 U/mL that continued increasing until 19 U/mL during the stationary phase, which is more than threefold higher than the optimal value predicted by the model, and 4.6-fold higher than the activity obtained using a whey medium without molasses, at the same culture time in shake flasks. From the beginning of the cultures, the expression is induced of recombinant ScAGal and native β-galactosidase and invertase, which allows the hydrolysis of raffinose, lactose and sucrose, respectively. The conversion of the sugar substrates during the bioreactor cultures was analyzed by HPLC and confirmed that the strain KGM21 consumes the 79.5% (w/v) raffinose and 98% (w/v) of the sum of lactose and sucrose. At the same time, 22.4 g/L ethanol was produced by fermentation and was then almost all consumed as carbon source, which is a behavior characteristic of the respire-fermentative metabolism of *K. lactis* [41]. A maximum peak of ethanol production appears at 24 h of culture and is quickly consumed without inhibiting the growth of the strain or the ScAGal production (Fig. 5b). Although it is described that, in industrial yeasts, glucose and fructose repress the metabolism of other less preferred sugars [29], we observed that KGM21 efficiently used the sugars in the mixture of whey and molasses and produced recombinant ScAGal. It has been reported that a *K. lactis* mutant in the gene *GAL1*, shows a decreased galactose consumption and, in consequence, an increased availability of this monosaccharide to induce the *LAC4* promoter [24]. A possibility for further research may be to test the hypothesis that this mutation increases ScAGal expression in KGM21.

Since the recombinant ScAGal is secreted to the extracellular medium, it has the advantage that it can be separated from the impurities of the production medium through tangential filtration membranes. In previous studies, we have successfully confirmed, using SDS-PAGE analysis, the concentration and purification of the recombinant protein with the tangential flow filtration technique (TFF, Millipore), suggesting that it was an efficient and effective process to avoid the use of purification systems that make the industrial production process more expensive [42]. In addition, we modified ScAGal to create a fusion protein with a poly-His tag to obtain a higher purity final product by affinity chromatography, if necessary.

On the other hand, the parameters of productivity (*Q*p) and yield from substrate of ScAGal (*Y*_*p*/s_) calculated from the cultures in YPL, YPW and the optimized whey-molasses medium are shown in Table 6. For the bioreactor culture, *Q*_*p*_ was 0.158 U/L/h, notably higher than the other shake flask cultures and *Y*_*p*/s_ was similar to the culture in shake flasks with YPW and also higher than the other cultures. The improved productivity in bioreactors is a common trait, generally attributed [4] to the fact that bioreactors are closed systems with a stricter control of aeration, agitation and temperature than other systems. Moreover, the mechanical agitation of bioreactors, compared to the orbital agitation of shake flasks, favors the continuous supply of oxygen to the cells and in consequence growth and bioproductions.

**Table 6:** ScAGal production parameters during fermentation by *K. lactis* KGM21

Culture	(U/mL)^a^	Y_p/s_^b^	Q_p_^c^
YPL flask	1.22 ± 0.20	0.061	0.010
YPW flask	4.07 ± 0.22	0.203	0.034
Optimized medium flask	6.08 ± 0.18	0.061	0.051
Optimized medium bioreactor	18.93 ± 1.26	0.190	0.158

^a^Extracellular ScAGal activity (μmol min/mL) to pH 4 and 40 °C

^b^ScAGal yield on substrate available in the production medium (Yp/s = Activity/g)

^c^ScAGal volumetric productivity in 120 h of culture (Qp = Activity/L h)

The proposed medium, based on whey and beet molasses, allowed the environmental innovation of the production process of ScAGal by *K. lactis* strains and, in turn, this could possibly be used in the heterologous production of other proteins with similar features to the object of study.

## Conclusions

We have engineered the *K. lactis* KGM21 strain harboring up to four copies of the gene coding for ScAGal (with its endogenous signal peptide) integrated at the *LAC4* chromosomal locus. An optimized mixture of milk whey and beet molasses turned out to be a cheap, sustainable and highly recommendable culture medium for high yield heterologous ScAGal production, directed to the extracellular medium by KGM21, thus allowing the valuation and ensuing reduction of the polluting impact of these agro-industrial wastes.

## Additional files

**Additional file 1: Figure S1.** Standard curves (A) and validation of the method 2^−ΔΔCt^ (B) by qPCR and *SYBGreen* detection. Three extractions of genomic DNA of the calibrator strain KGM28 and decimal serial dilutions were performed. *MEL1* (full circles), *TAF10* (empty circles), E = amplification efficacy. ΔCt_c_ (Ct_*MEL1,c*_ *− *Ct_*TAF10,c*_).

**Additional file 2: Figure S2.** Extracellular α-galactosidase activity produced by the strain KGM21 growing in lactose or cheese whey media, YPL and YPW, respectively. Data shown are average ± SD, N = 3.

**Additional file 3: Table S1.** ANOVA for response surface quadratic model.
