# Supplementary material for: Valuation of agro-industrial wastes as substrates for heterologous production of α-galactosidase
Source: Microb Cell Fact. 2018 Sep 3;17:137. doi: 10.1186/s12934-018-0988-6 (PMC6122717; doi:10.1186/s12934-018-0988-6)

**Additional File 1:**

**Figure S1:** Standard curves (A) and validation of the method 2^-ΔΔCt^ (B) by qPCR and *SYBGreen* detection. Three extractions of genomic DNA of the calibrator strain KGM28 and decimal serial dilutions were performed. *MEL1* (full circles), *TAF10* (empty circles), E = amplification efficacy. ΔCt_c_ (Ct*_MEL1,c_* - Ct*_TAF10,c_*).


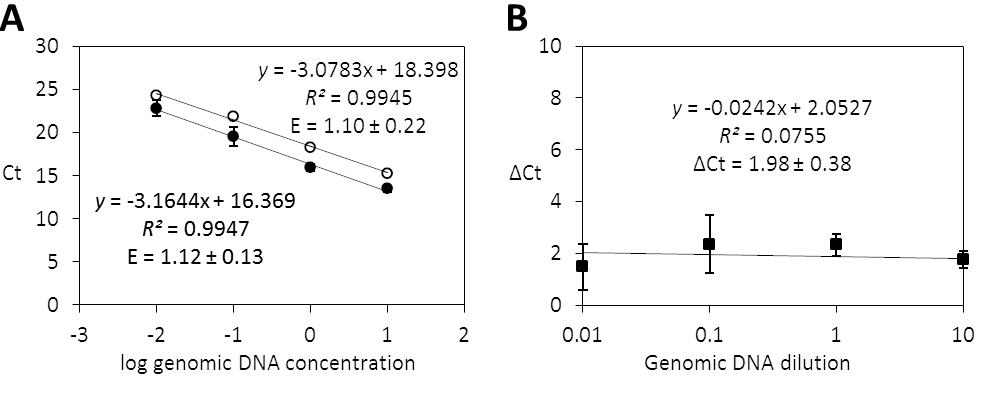

Supplement: Supplementary file 1 — Additional file 1: Figure S1. Standard curves (A) and validation of the method 2−ΔΔCt (B) by qPCR and SYBGreen detection. Three extractions of genomic DNA of the calibrator strain KGM28 and decimal serial dilutions were performed. MEL1 (full circles), TAF10 (empty circles), E = amplification efficacy. ΔCtc (CtMEL1,c − CtTAF10,c). [file 12934_2018_988_MOESM1_ESM.docx]
