# Supplementary material for: Valuation of agro-industrial wastes as substrates for heterologous production of α-galactosidase
Source: Microb Cell Fact. 2018 Sep 3;17:137. doi: 10.1186/s12934-018-0988-6 (PMC6122717; doi:10.1186/s12934-018-0988-6)
Supplement: Supplementary file 2 — Additional file 2: Figure S2. Extracellular α-galactosidase activity produced by the strain KGM21 growing in lactose or cheese whey media, YPL and YPW, respectively. Data shown are average ± SD, N = 3. [file 12934_2018_988_MOESM2_ESM.docx]

**Additional File 2:**

**Figure S2:** Extracellular α-galactosidase activity produced by the strain KGM21 growing in lactose or cheese whey media, YPL and YPW, respectively. Data shown are average ± SD, N=3.

**
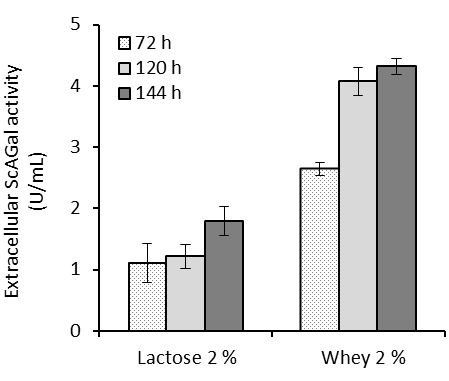
**
