# Supplementary material for: Valuation of agro-industrial wastes as substrates for heterologous production of α-galactosidase
Source: Microb Cell Fact. 2018 Sep 3;17:137. doi: 10.1186/s12934-018-0988-6 (PMC6122717; doi:10.1186/s12934-018-0988-6)
Supplement: Supplementary file 3 — Additional file 3: Table S1. ANOVA for response surface quadratic model. [file 12934_2018_988_MOESM3_ESM.docx]

**Additional File 3:**

**Table S1.** ANOVA for response surface quadratic model

| Effects^a^ |  | Sum of squares | Df^b^ | Mean square | F-ratio | *p*-value^c^ |
| --- | --- | --- | --- | --- | --- | --- |
| *x_1_* |  | 12.942 | 1 | 12.942 | 70.56 | 0.0035 |
| *x_2_* | | 0.016 | 1 | 0.016 | 0.09 | 0.7836 |
| *x_1_x_1_* | | 14.013 | 1 | 14.013 | 76.4 | 0.0032 |
| *x_1_x_2_* | | 1.150 | 1 | 1.150 | 6.27 | 0.0874 |
| *x_2_x_2_* |  | 0.803 | 1 | 0.803 | 4.38 | 0.1274 |
| Lack of Fit | | 1.013 | 3 | 0.338 | 1.84 | 0.3143 |
| Pure Error | | 0.550 | 3 | 0.183 |  |  |
| Total | | 34.857 | 11 |  |  |  |

*R^2^* = 95.51 %; adjusted *R^2^* = 91.77 %; standard error = 0.428; mean absolute error = 0.294.

^a^ Linear effects (*x_1_*, molasses; *x_2_*, whey), quadratic effects (*x_1_x_1_*; *x_2_x_2_*) and interaction effect (*x_1_x_2_*).

^b^ Df, Degrees of freedom.

^c^ *p* ≤ 0.05 denotes a statistically significant difference.
